# Influence of 3D Printing Topology by DMLS Method on Crack Propagation

**DOI:** 10.3390/ma14237483

**Published:** 2021-12-06

**Authors:** Karel Dvorak, Jana Dvorakova, Lucie Zarybnicka, Zdenek Horak

**Affiliations:** Department of Technical Studies, College of Polytechnics Jihlava, Tolsteho 16, 586 01 Jihlava, Czech Republic; jana.dvorakova@vspj.cz (J.D.); lucie.zarybnicka@vspj.cz (L.Z.); zdenek.horak@vspj.cz (Z.H.)

**Keywords:** AlSi10Mg, DMLS, printing topology, compact tension specimen, crack, cyclic loading, fatigue

## Abstract

The presented text deals with research into the influence of the printing layers’ orientation on crack propagation in an AlSi10Mg material specimen, produced by additive technology, using the Direct Metal Laser Sintering (DMLS) method. It is a method based on sintering and melting layers of powder material using a laser beam. The material specimen is presented as a Compact Tension test specimen and is printed in four different defined orientations (topology) of the printing layers—0°, 45°, 90°, and twice 90°. The normalized specimen is loaded cyclically, where the crack length is measured and recorded, and at the same time, the crack growth rate is determined. The evaluation of the experiment shows an apparent influence of the topology, which is essential especially for possible use in the design and technical preparation of the production of real machine parts in industrial practice. Simultaneously with the measurement results, other influencing factors are listed, especially product postprocessing and the measurement method used. The hypothesis of crack propagation using Computer Aided Engineering/Finite Element Method (CAE/FEM) simulation is also stated here based on the achieved results.

## 1. Introduction

Additive technology, representing a broad portfolio of production methods, is gradually becoming one of the conventional production processes [1]. In engineering industrial practice, technologies of metal 3D printing and Direct Metal Laser Sintering (DMLS) are mainly used [2,3,4]. With their characteristics, additively manufactured metal products approach equivalent materials processed by conventional casting, forming, and subsequent machining from cast and developed semi-finished products [5]. The advantage of additive technologies is fast technical production and the possibility of achieving free complex shapes [6]. By conventional technologies, there is a costly and time-consuming production of a tool for casting or forming or for the preparation and debugging of a program controlling the device’s path during machining. Three-dimensional printing technology is suitable due to its productivity, especially for a piece or small series production.

A typical example is the production of component prototypes within development, assembly, and testing. The advantage is also the possibility of the fast output of a complex spare part for the existing equipment, where the low productivity and cost of 3D printing are balanced by the rapid preparation of part production. The above-listed options are consistent with current trends in component development, where the virtual prototype plays a key role, represented by a 3D model created using a Computer Aided Design (CAD) tool. These procedures are currently an industry standard. Thus, a digital 3D model of the part is usually available at all stages of the product life cycle, which is directly or with minimal modifications sufficient for the technical preparation of part production by one of the 3D printing methods. During 3D printing, the product’s shape is modeled by adding material or solidifying the material in non-solid form. DMLS, which is the most suitable method for producing components of everyday engineering use, is based on the sintering of metal powder in layers by a laser beam. The resulting homogeneous structure corresponds to the material characteristics of the base material. In terms of visual aspects and the general compactness of the resulting body, the parts are identical. The essential factor for the practical use of the component is its mechanical properties, particularly the behavior of the material under mechanical stress corresponding to the functional characteristics of the part. One of the critical factors in the functionality, safety, and reliability of the design solution is the service life of the individual design elements, directly impacting the service life of the equipment as a whole. A typical factor of moving equipment, or equipment exposed to environmental conditions, is cyclic stress. This load can cause crack propagation in the material, leading to destruction and thus loss of functionality.

The subject of this research, AlSi10Mg, is a commonly used material processed by the DMLS method. This alloy has been used in the aerospace and automotive industry [7]. In general, it is a standardized, well machinable aluminum alloy used on low-strength machinery components, especially where low weight, acceptable corrosion characteristics, good availability, and easy machinability are required [8]. Due to various possible static and dynamic characteristics of specimens prepared by additive technologies, it is necessary to verify the testing of different types of structure orientation in 3D printing to choose the proper application and thus avoid the possibility of product rupture during its stress. Testing the mechanical properties of AlSi10Mg 3D-printed alloy is an up-to-date topic. More scientific publications are available regarding static testing [9,10,11,12] and dynamic testing [13,14]. It is also essential to test the mechanical properties regarding temperature during loading [15,16].

This research focuses on testing crack propagation in specimens prepared by DMLS technology using different types of specimen orientation during printing. In general, it is a critical parameter that affects mechanical properties, microstructural properties (e.g., porosity), printing time, etc. [17,18,19,20,21]. In this study, the characteristics of specimens after testing were also examined with a digital optical microscope for crack propagation analysis. This enables us to make judgments about the possibilities or influence of porosity.

The Computer Aided Engineering/Finite Element Method (CAE/FEM) simulation of internal stress is a vital pre-production step, which helps us to predict the behavior of materials under cyclic stress with respect to the process of 3D printing itself, where it is possible to choose the optimal production parameters. The study [22] looked at the application of crack propagation for a gear model because the contact conditions between real gear teeth near the pitting crack mouth were computed, and the pressured fluid lubricant penetration into the pitting crack was assumed. In this case, the material of Compact Tension (CT) specimens was the alloy 18CrNiMo7-6. Several critical studies [23,24] have been based on the use of CAD models at different loads to simulate the behavior of printed metal grids. In this study, an internal stress analysis was performed at the site of testing crack propagation through the material.

## 2. Materials and Methods

The tested material is chosen as a block semi-product, printed from AlSi10Mg (for material characteristics, see Table 1), by DMLS using a Renishaw AM400 machine (Renishaw, Wotton-under-Edge, UK). AlSi10Mg alloy contains 87.5 wt.% Al, 9–11 wt.% Si, 0.25–0.45 wt.% Mg and may contain a negligible number of elements such as Fe, N, O, Ti, Zn, Mn, Ni, Cu, Pb, Sn [25]. Semi-finished products are manufactured according to the following technological parameters (Table 2).

AlSi10Mg powder is made by elongated and spherical particles with a smooth surface consisting mainly of aluminum with silicon of mass fraction up to 10% and less than 1% of magnesium along with other minor elements. Precipitates of Mg and Si make the alloy stronger and harder at the same time [26].

Article [26], which deals directly with the parameters of the microstructure of the SLM method for the AlSi10Mg material, gives examples for various process parameters and directional orientations. In the series of pictures, the authors show maps of grain arrangement, including their sintering. The SEM method makes it possible to distinguish between the grains of the prescription of regular arrangements. The degree of regularity of the arrangement demonstrates the homogeneity of the structure. The homogeneous structure assumes little influence on the direction of crack propagation by random deflections during propagation. The unification of the tested material properties is an essential factor concerning the testing performance. The critical factor due to parameter topology and research hypotheses is the thickness of the layer, which is the primary factor of crack propagation due to its orientation. The layers represent a degree of material homogeneity, and the influence of direction on the crack propagation rate under cyclic loading is assumed.

**Figure 1:**
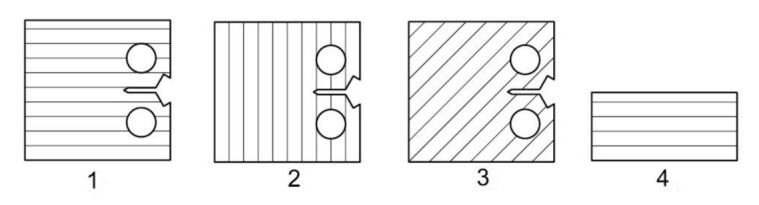
Sintering topology layout for all specimens: 1 = 0°; 2 = 90°; 3 = 45°; 4 = twice opposite 90°.

**Figure 2:**
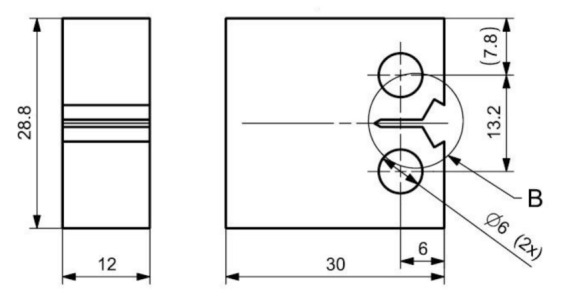
Compact tension testing specimen layout, dimensions in mm.

**Figure 3:**
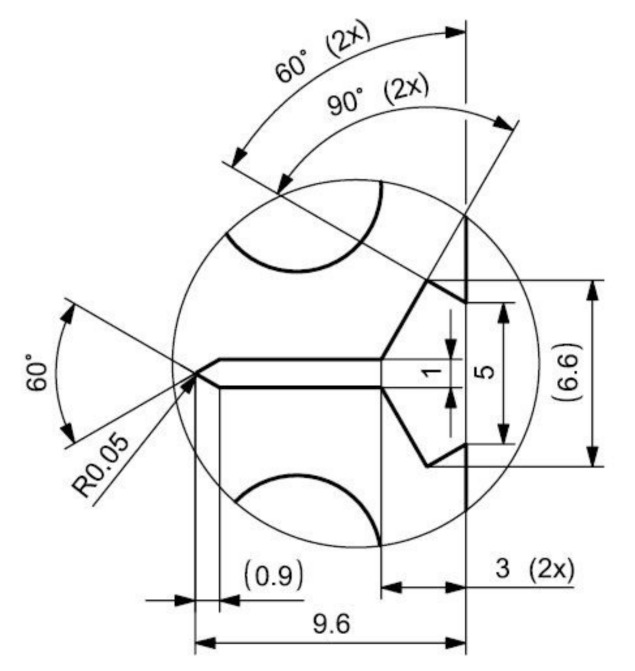
Crack detail of CT specimen, dimensions in mm.

Three-dimensional printing by specimen preparation represents the creation of a semi-finished product because metal 3D printing methods do not enable us to reach the exact dimensions required by the standard for crack propagation testing. In particular, the shape and dimensional accuracy of the initiated crack, requiring a radius of 0.05 mm at the beginning of the initiation, is necessary to achieve by the method of precision machining. Wire cutting (a wire with a diameter of 0.025 mm is used for cutting), milling, grinding, and polishing methods are used for the standard test specimens used in the experimental measurements. These procedures are a prerequisite for achieving the dimensional and shape characteristics prescribed by the standard CSN ISO 12108 [27]. At the same time, the quality of the surface is an essential factor, where minimal surface defects affect the speed of crack propagation. The quality of the surface and its determination directly affect the uncertainty of measurement results. The production of specimen semi-products is realized with the orientations of the printing layers, as shown in Figure 1 (drawing). Parallel lines represent respective orientations concerning the initiated crack. The realized printing topology, oriented to the position of the initiated crack and the particular direction of loading, is shown in Figure 1. The specimens were named, as can be seen in Figure 1. A drawing of the final shape of the 3D printing CT specimen is shown in Figure 2. The detail in Figure 3 shows the shape of the initiated crack corresponding to the standard and at the same time adapted to the available components of the test equipment, which is illustrated. According to the production method, the surface quality of the notch is max. *R*_a_ 0.4. The sides of the body are further ground to *R*_a_ 0.4. From each type of printed topology, 5 specimens are prepared and tested.

**Figure 4:**
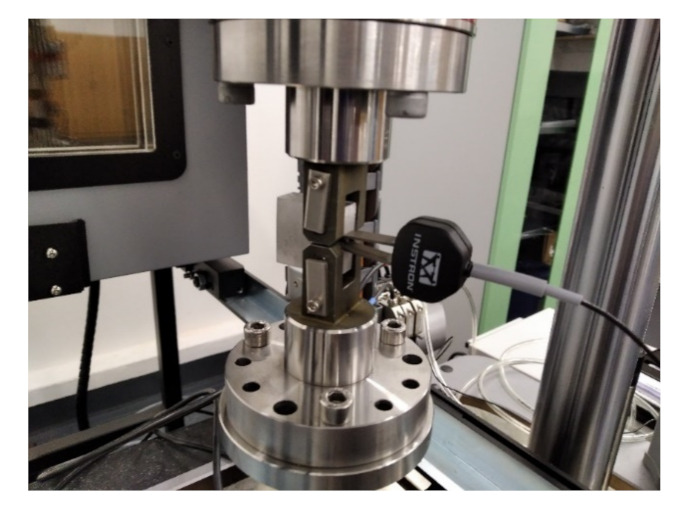
Testing fixture with CT specimen.

**Figure 5:**
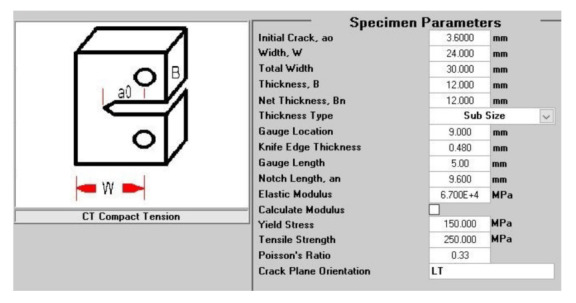
Setting specimen parameters.

Mechanical testing of CT specimens is performed on an ElectroPuls E 10,000 device (Instron, Norwood, MA, USA), using standard fixtures for clamping the specimen. The test method is based on a standard method CSN ISO 12,108 [27], adjusted according to the actual parameters of the specimen. The crack length measurement is performed indirectly by calculation from the parameters of the direct crack opening measurement. An electromechanical extensometer is used to measure the opening. The measuring system is shown in Figure 4. To define the measurement parameters and at the same time monitor the measurement process, the dadN software (Instron, Norwood, MA, USA) is used. The setting shows Table 3. Testing parameters of the specimen are shown in Figure 5. In addition to the geometrical characteristics of the test specimens, the input data for setting the initial test parameters are static values determined through a tensile test on standardized proportional test specimens in the form of rods of AlSi10Mg material to the standard CSN EN ISO 6892-1 [28].

The recording of measured values is performed continuously, during the measurement, into a data file, providing data in the * csv format, displayable and convertible into one of the other text or table formats. Conversion and evaluation are performed in MS Excel, as well as generating and publishing graphs of measured values.

The control and operation of the dynamic testing machine ElectroPuls E 10,000 are realized using the Instron Console software (Instron, Norwood, MA, USA) combined with electromechanical control console equipment. The testing force parameters are measured by a combined load cell. The determination of the control and evaluation parameters of the test is performed using the above-mentioned dadN application. Due to the execution and recording of the experiment only in one plane, the recording of torsional forces is not used. Torsion parameters are not even used for test control. Deformation characteristics are measured and recorded utilizing an electromechanical extensometer. Equipment accuracies and measurement uncertainties meet the requirements of the standard for the accuracy of the equipment used. Due to the performance of a standardized test on standard bodies, the appropriate standard fixtures are used simultaneously. In addition to the geometrical characteristics of the test specimens, the input data for setting the initial test parameters are static values determined using a tensile test on standardized proportional test specimens in the form of rods of identical material. In addition to the geometrical characteristics of the test specimens, the input data for setting the initial test parameters are static values determined through a tensile test on standardized proportional test specimens in the form of rods of AlSi10Mg material to the standard CSN EN ISO 6892-1 [28].

For the reliable performance of the experiment on a test device, a necessary step is to determine the material’s stiffness, which affects the subsequent reliable course of the individual cycles of dynamic loading. The adjustment is performed using a test specimen by an automatic adjustment cycle. By repeated adjustment of the test specimen, a linear stiffness of 15 kN/mm is found. The measurement is repeated five times for each of the four topologies. The results recorded are used for subsequent processing, evaluation, analysis, discussion, and defining conclusions.

The optodigital microscopy method was used to assess the shape consistency and verify the absence of defects on the surface of the test specimens. Optical imaging measurements were observed using a VHX-6000 digital optical microscope (Keyence, Osaka, Japan) to determine the roughness of the analyzed specimen. Five specimens were analyzed for each orientation before crack propagation testing. Specimens are checked from all directions. Particular attention is paid to the initiated crack’s surroundings to exclude the influence of initial defects on possible measurement results. This microscope using optical triangulation and having a resolution of approximately 1 µm was also used to observe specimens after mechanical testing. Magnification 50× for analyzing and monitoring specimens and a mapping function were used. This instrument re-constructed 3D maps of the surface after crack propagation testing for all specimens, magnification 100×.

CAD Siemens NX 11.0.2.7 software (Siemens, Washington, DC, USA) was used to develop drawing documentation and CAE Simcenter NX 11.0.2.7 (Siemens, Washington, DC, USA) to simulate the crack propagation process in specimens by CAE/FEM. CAE/FEM simulation verified the crack propagation hypothesis on a virtual prototype—a digital model of CT specimens, on an identical model, used to print a CT specimen. A curved, theoretically accurate model of the crack shape is used for wire cutting of the initiated crack on the resulting body. It is a static simulation, which aims to investigate stress conditions in the entire specimen volume, especially concerning other shape elements. The simulation refines possible considerations over the stress distribution in the specimen volume, mainly in the case of topology at an angle of 45°, demonstrably causing a crack deflection from the collinear direction. Accurate simulation of the loading force, symmetrical to the plane of the crack, also includes the distribution of the force in the holes for the pins of the jig. Loading force corresponds to the value used for cyclic loading. Due to the shape diversity of the specimen, the elements of the finite element network are determined in the shape of tetrahedrons. The size of the mesh elements is optimized for the overall size of the body to 1 mm. At the point of slight curvature of the top of the initiated crack, according to the 0.05 mm standard, the mesh is compacted to 0.025 mm. The loading force and the mounting of the body are chosen identically by the bearing in the load plane.

## 3. Results

The results of checking surface defects’ absence arising from production and subsequent processing are given in the form of the analysis of surface properties, expressed by the size of inequalities in Table 4. The uniformity of surface irregularities shows the absence of significant defects that could potentially affect the experiment. The table shows the average values for the particular topologies.

Results of the tensile test are listed in Table 5. Values of results are important to set the correct crack propagation test parameters. These are average values of individual topologies.

**Figure 6:**
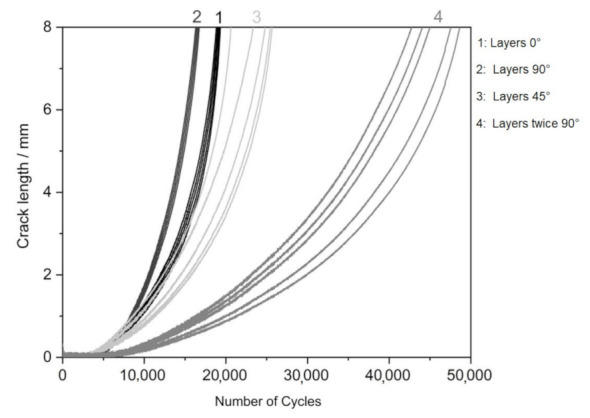
The number of loading cycles for all topology specimens.

Figure 6 shows the crack length as a function of the number of loading cycles. Fracture toughness (K_IC_) values of specimens have been written for all topologies in Table 6. This value means plane strain fracture toughness, which describes the minimum resistance a crack has to overcome to propagate through the surrounding material.

**Figure 7:**
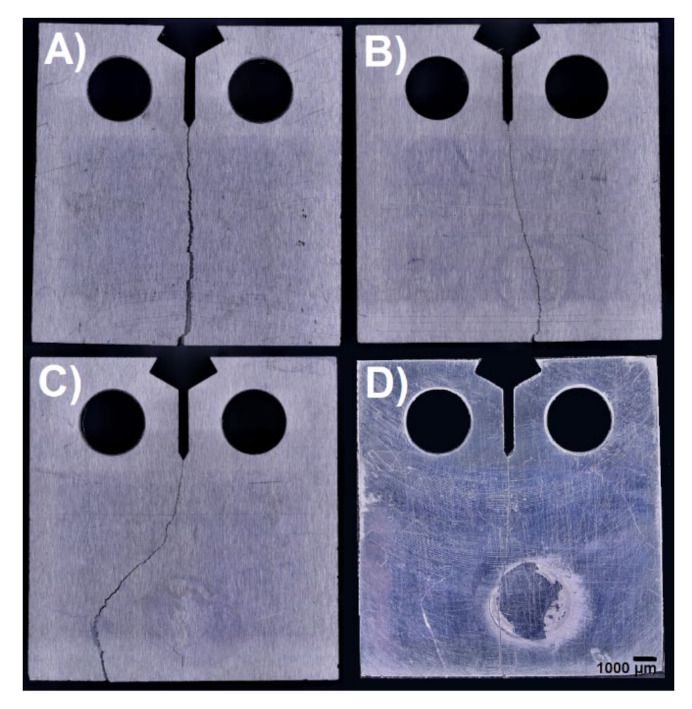
Crack propagation observation for all specimens: (**A**)—topology 1; (**B**)—topology 2; (**C**)—topology 3; (**D**)—topology 4.

**Figure 8:**
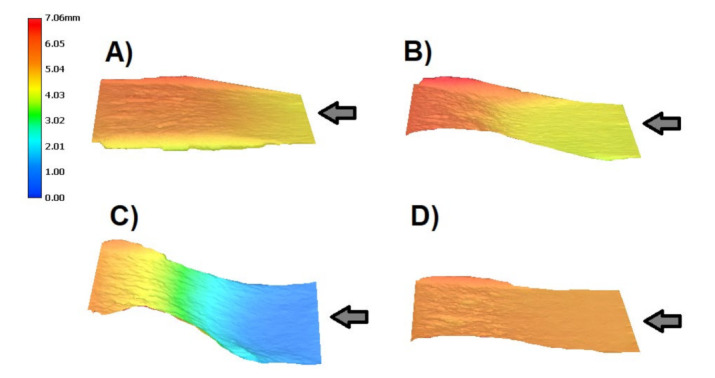
Three-dimensional models of crack propagation surface for all specimens, arrows directly show crack propagation: (**A**)—topology 1; (**B**)—topology 2; (**C**)—topology 3; (**D**)—topology 4.

Subsequently, the specimens were examined after mechanical stress using a digital optical microscope to monitor the propagation of cracks, which is affected by the print orientation of the specimens used and the internal porosity of the specimens. Figure 7 shows images of the specimens after said crack propagation testing. Three-dimensional models of crack propagation surfaces for all specimens are listed in Figure 8.

## 4. Discussion

The inspection of surface characteristics on a macroscale, especially the absence of significant cracks, showed the consistency of the surface of the tested bodies. These mechanisms depend on surface characteristics (texture, hardness) and test parameters (the applied load, temperature) [29]. Generally, the roughness of printed parts is up to 25 µm for this type of alloy produced by the 3D printing method [30]. Results for the tested specimens had roughness values in this range.

The effects of surface roughness on the low-cycle [31] and high-cycle fatigue [32] life are not a negligible parameter for a different type of metal alloy due to the crack initiation period [33,34,35]. Certainly, the behavior of materials during low-cycle fatigue will have an effect, as well as a heat treatment effect [32], anisotropy, grain characteristics, defects, surface roughness, and residual stresses [36,37].

The results show the influence of particular topologies. In general, the material must be considered less resistant to crack propagation, and in all cases, it is low-cycle fatigue. A significant difference is in topology 4, representing the printing layers perpendicular in two directions concerning the direction of the initiated crack. In this case, the crack length limit is reached after twice the number of loading cycles. The influence of the topology is also evident in the photographs of the finally broken specimens (see Figure 7), where the crack propagates almost rectilinearly for a parallel and perpendicular topology. The same result is evident for vertical topologies. With a layer inclination of 45°, crack propagation direction is deflected perpendicular to the individual layers, which corresponds to the results for specimen type 2. Topology 1 slows down crack propagation more than topology 2 with layers perpendicular to the crack propagation direction. From the tabulated values, the relative agreement of the individual measured specimens of the given topology is evident, also shown by the minor standard deviation in the topologies of the determined direction.

The topology at an angle of 45° shows better parameters than direct parallel and perpendicular topologies; the difference can be considered technically insignificant, especially to the significant variance of the measured values. The topology perpendicular in two axes to the crack propagation direction shows a twofold increase in the number of cycles to reach the comparative crack length, which can be considered technically significant despite the more considerable variance of the measured values. Specimens observed the best result with topology 3 and 4. The results were comparable. *R*_z_ was about 7.3 µm and *R*_a_ about 1.4 µm. The rest of the topology specimens had slightly worse results. Similar results for the polished specimens have been observed in the study [38].

The results of crack propagation testing show the influence of particular topologies. In general, the alloy AlSi10Mg material must be considered less resistant to crack propagation [39]. In all cases, it is low-cycle fatigue [31]. A significant difference is in topology 4, representing the printing layers perpendicular in two directions concerning the direction of the initiated crack. In this case, the crack length limit is reached after performing twice the number of loading cycles. The influence of the topology is also evident in Figure 8 of the finally broken specimens, where the crack propagates almost rectilinearly for a parallel and perpendicular topology.

The same result is evident for vertical topologies. With a layer inclination of 45°, the crack propagation direction is deflected perpendicular to the individual layers, corresponding to specimen type 2. Topology 1 slows down crack propagation more than topology 2 with layers perpendicular to the crack propagation direction. From the tabulated values, the relative agreement of the individual measured specimens of the given topology is evident, also shown by the minor standard deviation in the topologies of the determined direction. The topology at an angle of 45° shows better parameters than direct parallel and perpendicular topologies. The difference can be considered technically insignificant, especially to the large variance of the measured values. The topology perpendicular in two axes to the crack propagation direction shows a twofold increase in the number of cycles to reach the comparative crack length, which can be considered technically significant despite the more considerable variance of the measured values.

As part of crack propagation testing, the K_IC_ factor was also determined. The results are shown in Table 6. The values are comparable for specimens with topology 2, 3, and 4. Only the specimen with topology 1 shows twice the lower value. In the search, comparable results were confirmed for the specimen with topology 2. In our case, its value was 17.93 MPa·m^1/2^ compared with the literature [40] for the same orientation of the specimen when printing 12.9–15.2 MPa·m^1/2^. The results for the 45° orientation are noticeable when, in our case, the K_IC_ was published 17.93 MPa·m^1/2^ compared to the literature 13.6–16.2 MPa·m^1/2^ [40]. The images correlate with Figure 8. It can be seen here that the specimen with topology 3 has the most inhomogeneous effect.

Last but not least, it is necessary to mention the influence of the microstructure on the achieved results, as it is a very important parameter. Thijs et al. [41] studied the microstructure of AlSi10Mg manufactured using the Selective Laser Melting (SLM) process and observed three zones: the melt pool, melt pool boundary, and heat-affected zone. Manfredi et al. [42] also mentioned the same conclusion for the microstructural features of specimens developed by DMLS. It was proved in work [43] that after the production process by the SLM method, the struts on the microlattice structure remain on the surface. These objects due to um-melted or partially melted powder particles may play a significant role in crack initiation and propagation. The same observation was seen in [44]. AlRedha et al. [45] observed by metallography analysis using Scanning Electron Microscopy (SEM) that it is an evident interaction between the melt pool boundaries and micro-slipping in the course of the loading application. The melt pool boundaries could behave as more accessible pathways for crack propagation, resulting in a clear reduction in fracture toughness in specimens with cracks perpendicular to the build direction. This showed the evident anisotropic structure of SLM parts. The horizontally oriented specimen obtained the highest K_IC_ value. The observation indicated that build orientation has an important effect on fracture toughness.

**Figure 9:**
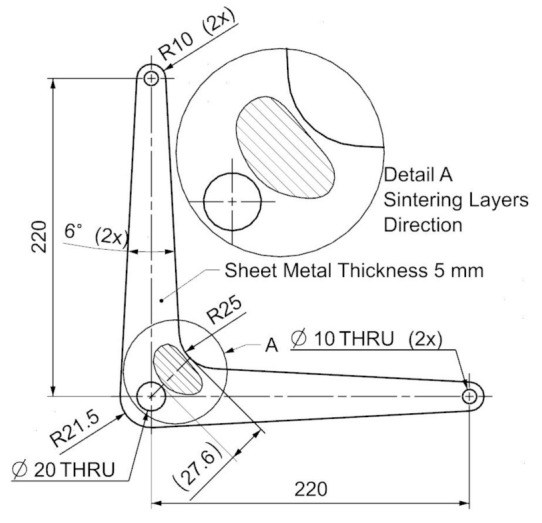
Applying the results of the experiment to the configuration of a real design solution.

**Figure 10:**
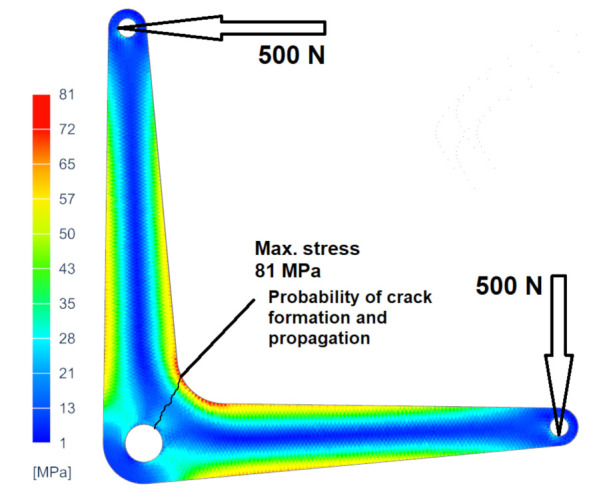
Result of the loaded lever simulation with the identification of the place of crack propagation in a functional loaded part manufactured by the DMLS method.

Figure 9 shows a specimen of the typical design application of the real product. There is a specimen of the lever, for example, as part of a control system used in small aircraft. In some cases, it is advantageous to use additive technology for prototyping or small series production. This enables the production of complex components, providing the required kinematic characteristics of the systems and at the same time easy assembly and operational collision-free operation of the system. The cost of the product is comparable or better than the technical preparation of production and subsequent production of the part, e.g., by numerical control (NC) machining. The lever shown is typically cyclically stressed. The calculation demonstrates the most significant load and the associated internal stress in the area marked in detail. Thus, it can be assumed, and it is also simulated or empirically verified, that there is local stress, crack formation, and destruction due to crack propagation. The example given is a critical situation if the hatching shown represents the projection of the printing layers. In this case, it is necessary to find the optimal print orientation, which is based on the experiment’s results, flat orientation. This example demonstrates the possible variability in the orientation of a part with known stress concerning the printing layers (Figure 10).

The practical application of measurement results is essential, especially in the design of relevant design solutions. These can often be significantly more complex components, where the appropriate orientation of the topology due to stress can significantly impact the resulting mechanical characteristics of the product.

## 5. Conclusions

Based on the results of experimental measurements and discussion, the influence of the topology of the component on the mechanical properties—the direction and speed of crack propagation under cyclic loading—was demonstrated. Contrary to the original hypothesis, when the influence of individual layers in the role of a damping factor for crack propagation across layers was expected, the results are opposite. The material can be considered to be significantly brittle, and the mechanism of crack propagation is, on the contrary, accelerated by the transverse printing layers. On the contrary, the crack propagation in the direction of the printing layers is attenuated due to the diffusion properties of the layers, which form a compact whole by consistent adhesion during melting. Nevertheless, the difference in the influence of perpendicular topologies for technical application can be considered insignificant. The effect of the orientation of the printing layers is evident in the topology, oriented 45° to the direction of the initiated crack and the applied cyclic load. This is clear in the crack shape for such oriented specimens. The practical application of the printed product based on the above conclusions does not lead to the need to consider the orientation when designing a mechanically stressed part. The influence of orientation perpendicular in two planes to the crack propagation direction can be considered a significant difference, shown by the specimen results according to topology 4. In practical application, after the analysis of component stress in the device assembly or separately, this factor can be regarded.

The example of practical application, given in the text, shows the possibility of analytical assessment and subsequent application of a typically cyclically stressed part. This is an example of the effectiveness of 3D printing technology, where it is possible to choose almost any orientation of the part during production. The starting point for producing a 3D printing product is a 3D model in the form of a virtual prototype. Simulation can be performed on model according to known boundary conditions to determine the stress distribution in the material structure and thus predict the location of the crack, including the direction of its propagation, which is supported by the results of 3D printing components.

In general, the AlSi10Mg material cannot be considered suitable for cyclically stressed components due to the considerably low cyclicity. This factor is important for the inclusion of material and additive manufacturing technology for the production of components that occasionally experience cyclic loading as part of their function over a small number of periods during the product’s life cycle. The advantage of additive technologies is the possibility of any orientation of the 3D printing body during production, which confirms the importance of research into the influence of the printing topology on the product’s mechanical properties.

## Figures and Tables

**Table 1:** Material characteristics [25].

Parameters	Values
Density	2.68 g/cm^3^
Thermal conductivity	30–190 W/mK
Melting range	570–590 °C
Tensile strength (XY)	442 ± 6 MPa
Tensile strength (Z)	417 ± 27 MPa

**Table 2:** Technological parameters for DMLS print.

Parameters	Values
Production speed	10 cm^3^/hod
Scanning speed	2000 mm/s
Positioning speed	7000 mm/s
Layer thickness	60 μm
Laser power	400 W

**Table 3:** Setting general testing parameters.

Parameters	Values/Information
Force range	2500 N
Stress ratio	0.1
Points/cycle	100
Waveform	sine
Frequency	10 Hz
Crack length	8.0 mm

**Table 4:** Roughness results for specimens before crack propagation measurement.

Specimen	*R*_a_/µm	*R*_z_/µm
Topology 1	1.83 ± 0.15	8.34 ± 1.09
Topology 2	1.52 ± 0.11	8.17 ± 0.98
Topology 3	1.42 ± 0.18	7.36 ± 0.57
Topology 4	1.37 ± 0.14	7.87 ± 0.68

**Table 5:** Tensile testing results.

Parameter	Value for Topology		
	1 (0°)	2 (45°)	3 (90°)
Tensile strength/MPa	449	447	331
Yield strength/MPa	262	254	199
Elongation at Break/%	4.6	4.7	2.4
Modulus of elasticity/GPa	69	67	61

**Table 6:** Tabulated overview of achieved numbers of cycles and results of fracture toughness (K_IC_) values of specimens.

Specimen	Type	The Average Number of Cycles	K_IC_/MPa·m^1/2^
Topology 1	0°	21 584 ± 209	8.95
Topology 2	90°	16 611 ± 273	17.93
Topology 3	45°	23 146 ± 2 182	17.93
Topology 4	Twice 90°	45 981 ± 2 291	20.10

## Data Availability

Data is contained within the article.
